# Review of Spider Silk Applications in Biomedical and Tissue Engineering

**DOI:** 10.3390/biomimetics9030169

**Published:** 2024-03-11

**Authors:** Marija Branković, Fatima Zivic, Nenad Grujovic, Ivan Stojadinovic, Strahinja Milenkovic, Nikola Kotorcevic

**Affiliations:** 1 Institute for Information Technologies, University of Kragujevac, Jovana Cvijića bb, 34000 Kragujevac, Serbia; marija.brankovic99@gmail.com; 2 Faculty of Engineering, University of Kragujevac, Liceja Knezevine Srbije 1A, 34000 Kragujevac, Serbia; gruja@kg.ac.rs (N.G.); strahinja.milenkovic@fink.rs (S.M.); nidzakotorcevic@gmail.com (N.K.); 3 Clinic for Orthopaedics and Traumatology, University Clinical Center, Zmaj Jovina 30, 34000 Kragujevac, Serbia; stojadinovic.ivan.78@gmail.com; 4 Faculty of Medical Sciences, University of Kragujevac, Svetozara Markovića 69, 34000 Kragujevac, Serbia

**Keywords:** spider silk, recombinant spider silk proteins, tissue engineering, drug delivery, bone and cartilage, ligament and muscle repair, repair of peripheral nerves, tissue-on-chip, organ-on-chip

## Abstract

This review will present the latest research related to the production and application of spider silk and silk-based materials in reconstructive and regenerative medicine and tissue engineering, with a focus on musculoskeletal tissues, and including skin regeneration and tissue repair of bone and cartilage, ligaments, muscle tissue, peripheral nerves, and artificial blood vessels. Natural spider silk synthesis is reviewed, and the further recombinant production of spider silk proteins. Research insights into possible spider silk structures, like fibers (1D), coatings (2D), and 3D constructs, including porous structures, hydrogels, and organ-on-chip designs, have been reviewed considering a design of bioactive materials for smart medical implants and drug delivery systems. Silk is one of the toughest natural materials, with high strain at failure and mechanical strength. Novel biomaterials with silk fibroin can mimic the tissue structure and promote regeneration and new tissue growth. Silk proteins are important in designing tissue-on-chip or organ-on-chip technologies and micro devices for the precise engineering of artificial tissues and organs, disease modeling, and the further selection of adequate medical treatments. Recent research indicates that silk (films, hydrogels, capsules, or liposomes coated with silk proteins) has the potential to provide controlled drug release at the target destination. However, even with clear advantages, there are still challenges that need further research, including clinical trials.

## 1. Introduction

The development of biomaterials aims for sustainable and biobased materials [1], including natural sustainable materials that can mimic tissue structures, such as silk-based biomaterials [2]. Silk-based biomaterials have been intensively studied for diverse applications in biomedical and tissue engineering [3,4,5,6,7]. The application of scaffolds with silk fibroin has been studied for bone scaffolding and ligament, cartilage, and tendon scaffolds, as well as for wound dressings and skin scaffolds [8,9,10,11,12]. Spider silk was selected and analyzed in this article as a natural sustainable biocompatible material, with excellent mechanical properties in comparison to other organic based materials, and a material which has been used for centuries in different applications. Biomedical applications of spider silk still account for a smaller part of its applications, whereas other industries have widely used it, even though there is a clear potential for its application in tissue engineering, including in musculoskeletal tissues, due to its favorable mechanical properties and other material properties that can further be customized in composite structures.

Spider silk has been used in many different applications, including tissue engineering [13,14]. In ancient times, spider silk was used to stop bleeding, where it served as an astringent [15,16]. The first clinical use of spider silk was in the 18th century, when it was used for suturing, whereas nowadays the application of silk as a biomaterial has been widely studied due to its excellent biocompatibility, high toughness, and ability to support tissue growth, especially for bone and ligament tissues [17,18]. Silk is one of the toughest natural materials and its fibers exhibit high strain at failure and very high mechanical strength [19]. Since its natural collection is limited, fabrication of the recombinant spider silk protein has begun [20]. Consequently, this technology has enabled modern biomedical applications [21]. All its excellent properties (very good mechanical characteristics, excellent biocompatibility, low density, and biodegradation) have shown high potential in tissue engineering [13]. The major clinical application of silk is in silk sutures, with a rather limited number of clinical uses in other medical applications, such as in cosmetics, wound dressing, breast reconstruction, and the treatment of gynecological conditions [22].

For musculoskeletal tissue regeneration (bone and cartilage tissue engineering), silk-based biomaterials offer a unique combination of properties and possibilities of molecular-level modifications and tailoring to the specific tissue scaffold [9,12,23,24]. Damage to the cartilage or degenerative conditions have influenced millions of patients, and in many cases joint replacements are the only possible treatments. However, these traditional treatments do not regenerate cartilage, just relieve patients of pain, unlike novel biomaterials with silk fibroin that can mimic the tissue structure and promote cartilage regeneration and new tissue growth [10]. Ligament tissue regeneration is complex and still under research, whereas silk-based materials show great promise [25]. For the repair of damaged intervertebral discs by using silk (hydrogels with silk and silk-based scaffolds), preclinical studies have shown excellent results [26], but clinical studies are still missing.

Research has shown that composites and polymer blends that contain silk have promising properties for hard tissue engineering, but also for soft tissues [8,27]. Smart and bioactive materials [28,29,30] have properties that enable them to interact with the surrounding tissue. With adequate stimuli, a controlled healing process can be started which results in repaired, new tissue. This is especially important for complex defects of the bones. Another advantage of silk-based biomaterials is their antibacterial and antimicrobial properties, but the possible tailoring of those effects in tissue scaffolds is still under study [31]. Bioactivity of the biomaterials used for tissue regeneration is complex [12], whereas biomineralization, or the formation of biominerals, is especially important for hard bone tissues. It has been shown that silk proteins can meditate that biomineralization process [32]. Self-healing biomaterial has been produced from spider silk [33], promising smart biomaterials. Further advancements can be expected with the development of new organ printing and biofabrication technologies [34,35], such as bioprinting with bio-inks containing silk fibroin [36,37], electrospun scaffolds containing silk fibroin [5,38,39], or 4D printing for biomedical applications [40].

This review will present the latest research related to the production and application of spider silk in reconstructive and regenerative medicine and tissue engineering, including nanomedicine and drug delivery systems.

## 2. Structure and Properties of Spider Silk

Spidroin proteins, composed of an N-terminal domain, repeated motifs, and a C-terminal domain, determine the hierarchical structure of spider silk [41]. Although spidroin I and II are believed to be the main silk proteins [42], the identification of more than 20 silk genes suggests that the number of spidroins in silk glands is higher than anticipated [43,44].

The primary amino acids in spider silk are glycine, alanine, and serine [16]. The method of forced spinning has revealed that the microstructure and tensile behavior of spider silk fibers are influenced by the silking force exerted on the dope [45]. Furthermore, the strength of spider silk is highly dependent on the size and orientation of the nanocrystals [46]. Rheological properties of natural silk are complex and shear thinning governs the behavior of the fiber during the spinning process [47]. On the other hand, ion electrodiffusion governs silk electrogelation—the formation of a gel structure from an aqueous silk fibroin solution with the presence of electricity [48].

**Figure 1:**
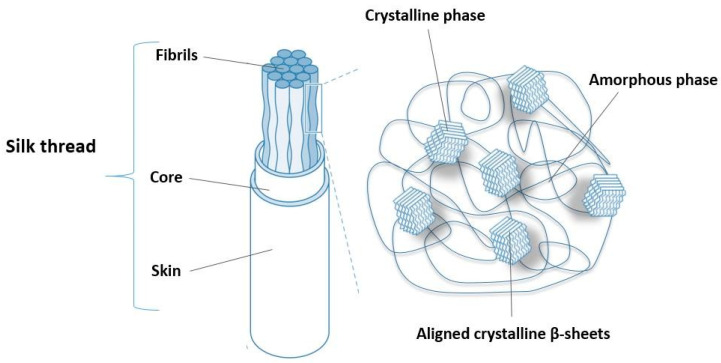
Structural organization of spider silk thread.

The microstructure of silk fibers is semi-crystalline, as shown in Figure 1, due to the presence of two phases: crystalline and amorphous (non-crystalline), as shown in [49]. The nanocrystalline phase is a result of the specific polypeptide secondary structure. Namely, in places with a high concentration of the amino acid alanine (polyalanine regions), several antiparallel β-sheets will form and group. These sheets are networked in an amorphous phase rich in glycine [50]. Weak hydrogen bonds are responsible for the superiority of this biopolymer in terms of its mechanical properties [51]. SEM and AFM imaging revealed that the silk thread (with a diameter of 4–5 μm) consists of many silk fibers with diameters in a range of 40–80 nm [46].

When cobweb fibers are exposed to high humidity or water, their length shortens significantly [52]. This phenomenon is called supercontraction, and the silk fibers are in the ground state [53]. The discovery of supercontraction made it possible to define different types of silk threads, that is, to establish a correlation between the mechanical properties of silk materials, the ecological niche, and the evolution of a given species [54]. In addition, this behavior of spider silk has been used to develop smart materials sensitive to temperature [55] and humidity. In this regard, the overall behavior of any silk fiber can be defined by the parameter α* that represents experimental magnitude that is determined from the reference Major Ampullate Silk (MAS) fibers [56].

## 3. Natural Synthesis of Spider Fibers

Spiders are cold-blooded organisms (poikilotherms) that can spin silk fibers with very high mechanical strength and toughness [57]. The temperature in their environment affects the speed of spinning of the web, and thus its mechanical and structural properties [58]. Likewise, silk varies widely in composition, depending on the specific source (spiders produce silk using seven different types of glands) [59].

Depending on life needs, spiders produce seven silk types (Table 1 and Table 2) [60], thanks to the various silk glands located at the rear end of the abdomen [61]. These types have different properties depending on whether they serve as a shelter, a means of catching prey, part of the love game, or as a particular thread the spider uses to escape in case of danger. Among them, “dragline” silk has been investigated in the most detail [13].

Major ampullate fibers have very high tensile strength and toughness (Table 3). Consequently, they form the frame and radially distributed supports of the spider web [13]. Their structure is layered, and each of the layers has a different role, e.g., the transmission of pheromones and recognition of gender and species, protection against microorganisms, and protection against damage by physicochemical agents or mechanical support. Generally, spider silk consists of at least two types of proteins: proline-free spidroins (MaSp1) and proline-rich spidroins (MaSp2) [62]. In addition to differences in the content of this amino acid, the spidroins also differ in hydropathicity. The MaSp1 class is hydrophobic, and the MaSp2 is predominantly hydrophilic [13].

Within the Major Ampullate (MA) gland, we distinguish four regions:

- The “tail” zone, which is responsible for the synthesis and secretion of spider web proteins;
- Lumen (bag) used for protein accumulation;
- Fiber alignment channel;
- Output for final fiber production.

As proteins travel from the lumen along the channel, they undergo elongation, promoting hydrophobic and hydrogen-bonding interactions. This is followed by the alignment of the proteins in the solution, resulting in stiffer and stronger fibers. Finally, the cobweb, excreted in liquid form, hardens very quickly in contact with air.

However, the collection of natural spider silk does not yield significant quantities. According to the Animal Welfare Act and European directives, animal stress is reduced to an absolute minimum, with no consequences during the collection procedure. For this purpose, spiders were fixed with gauze and needles on Styrofoam without anesthesia [63]. MA silk was isolated from the major ampullary glands using forceps. In this method, a mechanical stimulus is sufficient to initiate spider silk production, and the collection continues until the animal becomes distressed [63]. In each orb-weaver spider, the silk composition varies depending on the MaSp1 and MaSp2 spidroin content. For instance, Nephila claviceps has 81% MaSp1 and 19% MaSp2, while Argiope aurantia has 41% MaSp1 and 59% MaSp2 [7].

**Table 1:** Different types of spider silk (fibers).

**Major Ampullate Silk**	Major ampullate silk is produced in the main ampullary glands. These fibers serve to allow escape from predators. Also, they are used for the web’s outer rim and spokes. In this way, the other threads can be attached to them. They have a strength five times greater than steel and three times greater than Kevlar [59].
**Minor Ampullate Silk**	Minor ampullate silk is produced in the secondary ampullary glands. It has a role in the spiral formation of the network. Unlike MA fibers, it does not contain proline. Also, it has a reduced content of glutamate [59].
**Flagelliform Silk**	Capture-spiral (flagelliform) silk is produced in the flagelliform glands. It is used for catching prey [59].
**Tubiliform Silk**	Tubiliform (cylindriform) silk is produced in the tubiliform (cylindriform) glands. It is used for protective egg sacs [59].
**Aciniform Silk**	Aciniform silk is produced in the aciniform glands. It is a wrapping silk usedfor the immobilization of prey [64].
**Pyriform Silk**	Pyriform silk is produced in the pyriform glands. It functions like a glue, and connects the web to different materials [65].
**Aggregate Silk**	Aggregate silk is made in the aggregate glands. It produces aqueous gluey substances, making the capture threads sticky [66].

**Table 2:** Function and composition of different types of spider silk obtained from [16] under a Creative Commons License Type, CC BY 4.0.

Glands	Type of Spider Silk	Composition
Aggregate	Aqueous cement	ASG1, ASG2
Pyriform	Core fiber of capture spiral	PySp1, PySp2
Tubuliform	Egg-case silk	TuSp1, ECP-1, ECP-2
Flagelliform	Spiral silk	Flag
Aciniform	Capture silk	AcSp1
Minor ampullate	Dragline silk, framework silk	MiSp1, MiSp2
Major ampullate	Dragline silk, framework silk, radial silk	MaSp1, MaSp2

**Table 3:** Comparative presentation of the mechanical properties of spider silk and other fibers.

Material	Tensile Strength (Mpa)	Elongation (%)	Toughness (kJ/kg)
Dragline (MA) silk	4000	35	400
Silkworm silk	600	20	60
Kevlar 49	3600	5	30
Ligament	150	5	5
Bone	160	3	3

Spiders produce silk naturally in the form of fibers. However, silk produced through recombinant techniques involves the extraction of spider silk proteins in the form of powder. This technique allows for the combination of spider silk proteins with various materials to create fibers with different mechanical and structural properties. Although using expression systems makes recombinant production cost-effective, the process of purifying spider silk protein powder is both time-consuming and expensive [67].

## 4. Recombinant Production of Spider Silk

Biotechnological production has opened new approaches to produce spider silk proteins from other sources like bacteria, plants, yeasts, cells, or animals, to provide cost-efficient and stable fabrication [68]. The most commonly used proteins are derived from sequences isolated from the species *Nephila clavipes* and *Araneus diadematus* [13].

Recombinant protein production involves the following steps [69]:

- Determining the sequence of nucleotides in natural DNA (isolation of the desired sequence that encodes the target protein);
- Designing recombinant DNA;
- Selection of the vector that will enable the transmission of the desired sequence;
- Transmission of the vector into the host’s organism (bacteria, yeast, plants, insect cells, mammalian cells, and transgenic animals);
- Cultivation/production of proteins in the host organism;
- Isolation of the obtained proteins.

**Figure 2:**
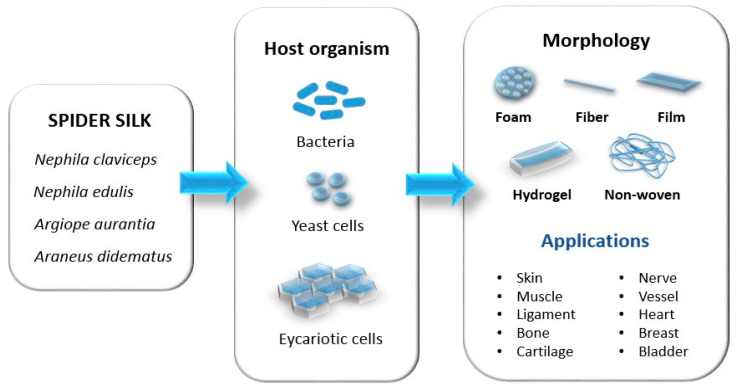
Production of recombinant spider silk proteins, sources, and possible biomaterials. Adapted from “Spider Silk for Tissue Engineering Applications” under Creative Common CC BY 4.0 license; the original work can be found at [13].

A schematic representation of the recombinant spider silk proteins’ production, sources, and possible biomaterials is shown in Figure 2.

Organisms used as hosts for silk protein fabrication can be different and the most commonly used hosts, as shown in Figure 2, are bacteria like *Escherichia coli (E. coli*), yeasts like *Pichia pastoris*, and mammalian cells like hamster kidney cells, but insect cells can also be used, like *Spodoptera frugiperda. E. coli* proved to be the most suitable host due to a high density of cells that grow fast and can be easily transformed. Biotechnological manipulation and production allow the modification and improvement of silk characteristics [70].

Significant progress has been made in recombinant spider silk production, but further improvements are necessary to overcome the main challenges of high investments and small product yields [67]. Additionally, the commercial use of recombinant spider silk has been limited due to the inability to produce spidroins at their natural size. For example, transgenic mammals and insects have the potential to produce larger proteins, which are easier to purify. However, growing such organisms is costly, and the yields are typically low. On the other hand, *E. coli* is relatively affordable to cultivate, but it is not efficient in expressing larger spidroins [71].

It is a common misunderstanding that the choice of host organism for producing recombinant spider silk only depends on the intended application. Advancements in bioinformatics have facilitated a more thorough examination of the distinct repetitive segments that significantly impact the structure, properties, and function of spidroin. These insights can enhance the efficiency of current host platforms and stimulate the development of novel production techniques to cater to specific production requirements [71].

Producing spider silk proteins using bacteria is a challenging task due to the high molecular weight of the proteins and their long repetitive regions, containing high levels of glycine and alanine. However, recent studies using *E. coli* have tried to overcome this issue by using alternative methods that involve the intein system to assemble protein subunits. This has led to the creation of chimeric fibers with impressive mechanical properties [72,73]. Additionally, other bacteria like *Corynebacterium glutamicum* [74], *Salmonella typhimurium* [75], and *Rhodovulum sulfidophilum* [76] have also been studied for their potential benefits in recombinant production. Among yeasts, *Pichia pastoris* [77] and *Saccharomyces cerevisiae* [78] successfully expressed these proteins.

It is not practical to use cell cultures from mammals or insects to produce structural proteins. However, scientists have had some success in expressing spider silk protein in certain types of cells, such as bovine mammary epithelial cells, hamster kidney cells [79], African green monkey kidney cells [80], and silkworm neuronal cells [81].

The use of transgenic plants represents an economical approach to produce large recombinant proteins, but in low yields [82]. In this context, various plants have been studied for the production of recombinant silk proteins, such as the species *Nicotiana tabacum* [83,84], *Solanum tuberosum* [85], *Medicago sativa* [86], and representatives of the genus Arabidopsis [87]. Also, transgenic animals have played a significant role in the production of recombinant spider silk through genetic engineering. Among them, transgenic silkworms have proven to be the most effective due to their ability to spin fibers [88]. Transgenic goats produced milk containing recombinant spider silk proteins, up to 0.5 g/L. Purified protein powder was obtained from such milk using special filtration techniques [89]. However, attempts to express recombinant spider silk proteins in sheep hair follicles have been unsuccessful [90].

## 5. Spider Silk Structures

Tissue regeneration is the process of tissue renewal and regrowth. Biomaterials, together with cells and bioactive factors, are the “building blocks” for making structures that resemble living tissues, through their combination in specific conditions. Appropriate substrates and scaffolds for tissue engineering must support the activity of cells in terms of their adhesion, migration, proliferation, and differentiation. Today, three-dimensional porous systems are the most adequate for cellular nutrition, respiration, and metabolism [13]. The level of porosity and the size of the pores has critical influence on the formation of the bone and bone regeneration [91]. The degradation of scaffolds over time allows space for the growth of new tissue, while adequate scaffold strength supports already-existing tissues [13].

### 5.1. Spider Silk in Fiber form (1D)

In Kuhbier et al.’s (2010) research, the process for obtaining cobweb fibers is detailed [92]. Once collected, the fibers are combined into bundles of 60–120 individual fibers and stored on large polypropylene tubes. These tubes are kept in an environment suitable for spiders for 6 months. After this period, spider silk sutures are produced using a miniature knitting machine and stored until biomechanical testing is conducted [93]. Sutures are commonly used to repair musculoskeletal tissue and therefore need to be able to withstand continuous mechanical stress. To test this, spider silk sutures and commercial Prolene® 6-0 sutures (ProNorth Medical, online store, Canada) were subjected to 1000 stretching cycles. The study results revealed that spider silk was not affected by continuous use, while the failure load of Prolene® 6-0 was significantly reduced. Furthermore, Prolene® showed a 24% ± 1.9 increase in strain, while spider silk only showed an increase of 7.2% ± 0.48 [94].

Spider silk fibers are attractive due to their mechanical strength and stability, biocompatibility, and good surface-to-volume ratio. On that note, MA silk has been investigated as a potential treatment agent for tendon ruptures [12,23,94]. Using ordinary sutures, the rate of successful regeneration of tendons was low, and an infection or reaction of the organism was often caused, so there is an incompatibility with the mechanical properties of the tendon tissue [13].

In another study, parallel silk fibers were studied as a substrate for developing human neurons. The neuronal cell bodies came into contact with the spider silk fibers, and over four weeks, ganglion-like structures formed [95].

Also, the woven spider silk was used in skin reconstruction. After sterilization, two weeks of fibroblast cultivation, and the addition of keratinocytes, a two-layer skin model was formed [96]. Furthermore, these fibers have been tested in preclinical models related to the reconstruction of the bladder [97].

### 5.2. Spider Silk Coatings (2D)

Spider silk coatings have been studied for different biomaterial applications, especially biological response, physicochemical characterization, and parameters that determine the final coating properties [98]. These coatings can be customized from aspects of different properties aiming to support better scaffolds in tissue engineering and natural-based materials as coatings on implants, but also for the development of biosensors [99] and to serve in surface functionalization for bioactive materials [100,101]. The design of new thin films based on spider silk showed possibilities of tailoring morphologies and hydrophobicity, as very important properties of the biomaterial surface, thus opening wide application areas for silk-based coatings and thin films [102]. Drug delivery systems can also utilize the possibility to customize spider-silk-coating properties [103].

The use of a coating is commonly considered to be a surface modification technique in the case of issues with surface responses. For example, the surface of a silicone breast implant can be coated with a thin film of spider silk to prevent fibrous tissue formation, which is a common issue [104]. Furthermore, significant improvement in the biocompatibility of the implant was observed, as well as reduced postoperative inflammation [104]. Films are made by dipping the test samples into a silk protein solution three times for 120 s. After each dip, the samples are left to dry for 300 s at room temperature. The formation of the β-sheet is induced by treating the films with KH2PO4 (1M solution) for 120 s, followed by air drying for 120 s. Finally, all test samples are rinsed with a 0.9% *w*/*v* NaCl solution [104]. During the initial stage of inflammation, certain types of cells such as CD4+, CD8+, CD68+, and TGFß1+ cells, along with pro-inflammatory cytokines IL-6 and TNF-a, are likely to appear. Compared to uncoated implants, silicone implants coated with spider silk showed a significantly lower expression of all the mentioned factors [104]. Spider silk coatings on different polymer catheters showed low cell adhesion, and almost no response from the surrounding tissue, and with good biocompatibility this can be a good coating material for catheters [105]. Silk structures have inspired the design of thin films to serve as bioelectronic interfaces (interfaces between tissue and electronics), as a very significant element for further development of flexible bioelectronics, including shape-adaptive biomaterials [106].

### 5.3. Three-Dimensional Constructs

Porous biomaterials have been proven to be the best candidates for guided cell growth and the better acceptance of implants by the body, or for tissue engineering and bone regeneration [91]. A porous foam made from recombinant protein spider silk pNSR-16, with a pore size of 250~350 nm, showed that fibroblasts form cell-rich zones on the surface and inside the structure [107]. To create the foams, a mixture of spider silk solution and granular NaCl (used as a porogen) was prepared and placed in a container. The mixture was heated in an oven at a temperature range of 55~60 °C for 30 min. As a result, NaCl-silk protein blocks were obtained. These blocks were first soaked in ethanol to induce a β-sheet structural transition and then in distilled water to extract the salt [107]. The cytotoxicity of the scaffold was tested in vitro on NIH-3T3 cells. Within six days, the entire surface of the scaffold was completely covered with cells, with very little space between them. Each subsequent day, the number of cells increased, clearly confirming that NIH-3T3 cells can easily attach, grow unhindered, and secrete the extracellular matrix on the pNSR-16 recombinant spider silk protein scaffold [107]. Likewise, the foam made of recombinant protein 4RepCT enabled the human stem cells to integrate and deploy [13]. The differentiation of human mesenchymal cells into adipocyte lineage was also studied using porous foams. This research has shown positive results because of lipid droplets commonly found in adipocytes (fat cells) [108]. Resorbable membranes have been studied for controlled bone regeneration [109].

In the tissue engineering of soft tissues, scaffolds need to support the surrounding tissue with adequate mechanical strength to enable cell growth and proliferation, and in cases of biodegradable implants, the rate of biodegradation needs to be controlled in accordance with the surrounding tissues. Porosity and other properties of the silk-based foams can be tailored to desired properties according to the surrounding tissues, even up to highly porous scaffolds [110]. Porous materials with a high toughness of incorporated fibers can absorb a high quantity of mechanical energy [18], which recommends them for the construction of bone tissue scaffolds.

Hydrogels are polymer networks with a water content above 95% and a high swelling rate [111]. Spider silk hydrogels can be used in a biofabrication combined with living cells to create a hierarchical tissue-like structure [112]. Cytocompatible bioinks suitable for cells and 3D printing are currently challenging to develop [113]. In this regard, hydrogels from recombinant spider silk proteins have shown promising properties [13].

The protein eADF4(C16) is an engineered version of the spider silk protein that mimics the repetitive part of the dragline silk fibroin ADF4 found in the garden cross spider (*Araneus diadematus*). The optimal method for creating hydrogels from eADF4(C16) involves the dialysis of low-concentration protein solutions out of 6 M guanidinium thiocyanate (GdmSCN) into 10 mM Tris/HCl (pH of 7.5), followed by dialysis against a PEG solution. Hydrogels obtained in this way are often combined with living cells, such as fibroblasts, to create tissue-like structures [13].

**Figure 3:**
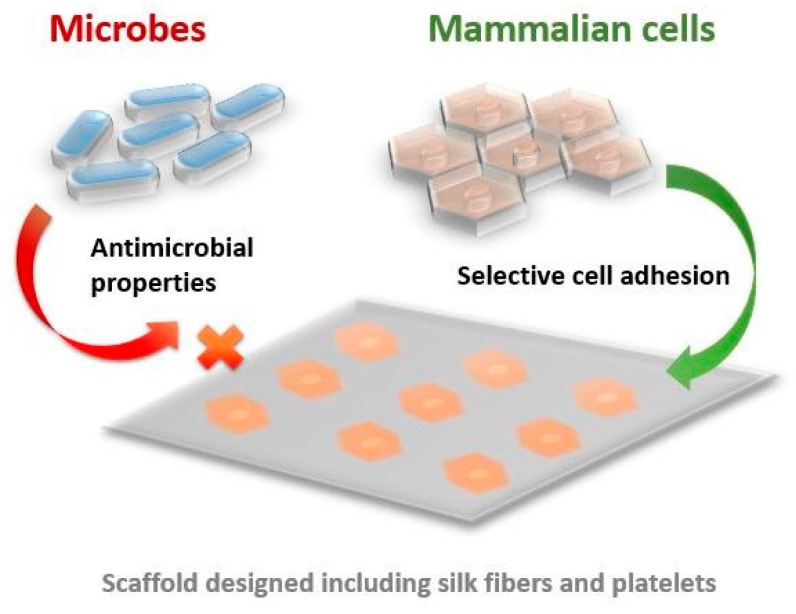
Antimicrobial properties of spider silk.

Antimicrobial hydrogels based on hyaluronic acid and spider silk (HA/Ss) are one more interesting example. These hydrogels are made by dissolving hyaluronic acid in 0.1 mol/L MES (4-(N-morpholino)ethanesulfonic acid) and mixing it with spider silk dissolved in trifluoroacetic acid (TFA). The resulting HA/Ss mixture is then polymerized using 100 mM NHS (N-Hydroxysuccinimide) and 100 mM EDC [1-ethyl-3-(3-dimethylaminopropyl) carbodiimide hydrochloride]) Exceptional antimicrobial activity against both Gram-positive (*Micrococcus sulfuricum*) and Gram-negative *E. coli* bacteria is due to the presence of hyaluronic acid, which inhibits protein synthesis, and spider silk, which prevents bacterial adhesion [114]. Antimicrobial properties of spider silk are shown in Figure 3.

Hydrogel scaffolds can be customized to enable the tailoring and control of cell migration, proliferation, and differentiation by controlling the transport of nutrients and oxygen and by mimicking the surrounding natural tissue [115]. A comprehensive review of hydrogels with silk fibroin aimed for tissue engineering and drug delivery systems is given in [116,117]. Hydrogels with a combination of silk fibroin with other bioactive compounds like alginate have shown promising properties for wound healing [118]. Hydrogel-based embolic agents for transcatheter arterial embolization showed very good results in endovascular embolization [119]. The sincorporation of smart properties has provoked research interest in making hydrogels that can sense external stimulus, and hence can be used in medical diagnostics [120] or drug delivery systems [121].

Considering the development of advanced technologies, such as the 3D printing of gels [122], possibilities for customized solutions in which silk fibroin can be modified and combined with other materials to form printable hydrogels [123] and functional silk protein hydrogels [124] are realistically wide-ranging.

Hydrogels with silk can be utilized for drug delivery in osteoarthritis treatment [125,126] or can act as self-healing scaffolds for bone regeneration, through the filling of bone defects [127]. Self-healing silk hydrogel can also be used as a flexible strain sensor with self-adhesive property [128] that can be used for the development of wearables, with immense significance, as well, for medical diagnostics. In cartilage tissue engineering, biomimetic hydrogels have shown excellent properties for constructing biomimetic scaffolds that support regeneration, but they still have challenges regarding mechanical properties [129]. Recent research has studied possibilities of injectable composite hydrogels with silk fibroin in spinal treatments [130,131].

**Figure 4:**
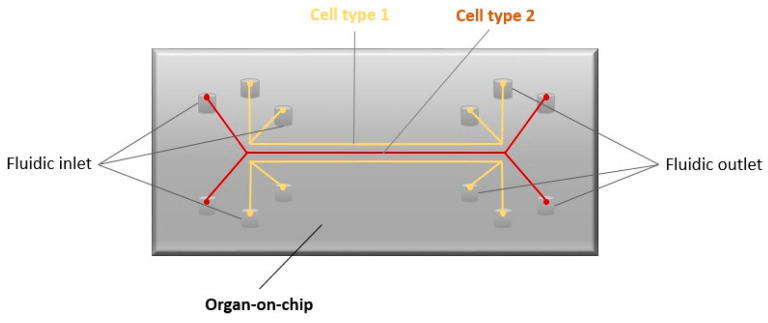
Organ-on-chip.

Recent advances in additive manufacturing and the availability of stem cells have opened up exciting possibilities for the development of bionic organs, including tissue-on-chip and organ-on-chip designs [132]. Organ-on-chip, as shown in Figure 4, contains microdevices with cells, microfluidics, and extracellular matrix scaffolds and can replicate microtissue and associated physiological processes, making it an ideal substitute for animal models in preclinical trials [133]. Several such chips have already been designed, including the 3D-printed ACL-ON-CHIP [134], which is used for the precise engineering of ligaments and their surrounding environment. Designing these micro-devices is important to understand the in vivo responses of silk proteins [135].

## 6. Applications in Tissue Engineering

### 6.1. Skin Regeneration

Engineered scaffolds of connecting tissues and skin have greatly benefited from all the excellent properties of silk-based materials, including the development of wound dressings with silk compounds [3,5,11]. One study presented the application of materials from recombinant spider silk for wound dressings on models of rats. The experiment used silk from pNSR-16 and pNSR-32 protein in second-degree burns. Interestingly, the materials from recombinant proteins of spider silk showed much better results than the control group (collagen), related to the skin regeneration [136]. Compared to the positive and negative control groups, wound healing was significantly faster in the group treated with recombinant spider silk proteins (*p* < 0.01). On the 14th day after treatment, in the sample with pNSR-16 and pNSR-32, the wound tissue was regenerated by newly formed, densely distributed epidermal cells, with a thickening of the subcutaneous tissue and a decrease in inflammatory cells. On the other hand, control groups did not show complete recovery even after the 21st day [136]. To improve cell adhesion, scientists modified silk with fibronectin [13]. In the case of third-degree burns in the animal model, the microporous dressing showed accelerated healing compared to a commercially available DuoDERM patch. Furthermore, histological analyses confirmed that wound healing in animals was accompanied by vascularization [13].

### 6.2. Bone and Cartilage Tissue Repair

**Figure 5:**
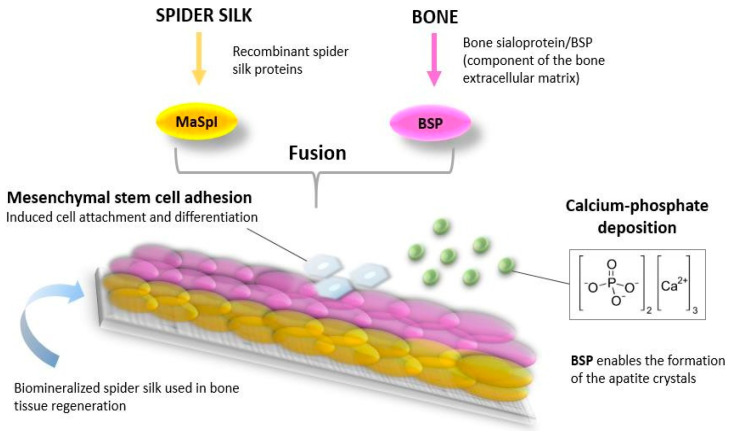
Use of spider silk protein (major ampullate spidroin protein) in the fabrication of scaffolds for bone tissue regeneration.

The bone naturally consists of inorganic (predominantly calcium-phosphate) and organic substances (predominantly collagen). Accordingly, materials made from recombinant spider silk proteins can be biomineralized and used in bone regeneration [32]. Silk fibroin can be applied for bone tissue growth, proved by both in vitro and in vivo tests [8]. The MaSp1 class of proteins combined with the BSP fusion protein induce calcium-phosphate deposition but also a good adhesion of mesenchymal stem cells (it significantly contributed to their differentiation) and the noticeable synthesis of type 2 collagen in cartilage cells (Figure 5) [137].

**Figure 6:**
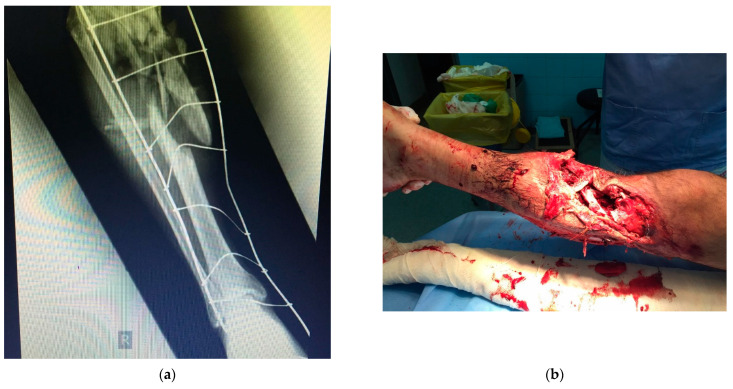
Open multifragmented fracture of the lower leg. Bone graft was applied. (**a**) radiography image of the fracture; (**b**) real image of the complex fracture before the surgical procedure.

**Figure 7:**
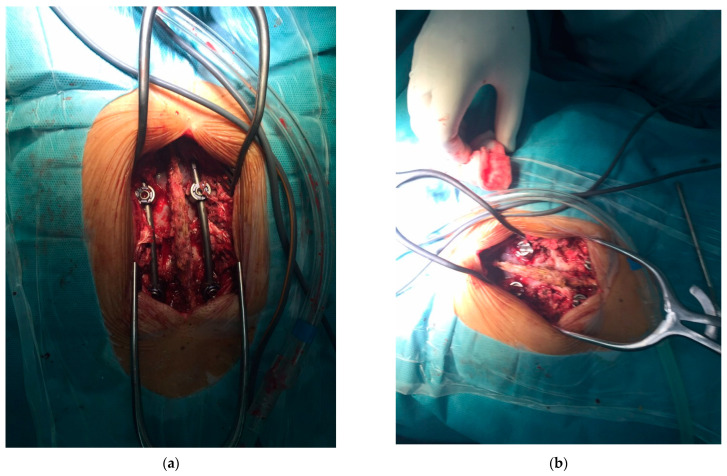
Intraoperative image after stabilization of spinal fracture. After positioning of the transpedicular screws, bone grafts are applied for better final fusion. (**a**,**b**) two views of the stabilization method during the surgical procedure, with prepared bone graft shown in the right image.

Currently used bone grafts are commonly positioned to support bone healing in the case of large fractures, such as the one shown in Figure 6, or to provide the additional stabilization of spinal disks in case of spine fractures, as shown in Figure 7. For multifragmented fractures (Figure 6), bone grafts must be applied to support the bone healing process and the use of advanced silk-based scaffolds should enable active bone grafting, even though it is not part of clinical practice yet. Better fusion provided by the bone grafts for complex spinal fractures (Figure 7) also needs bioactive materials, and this is yet another example where silk-based scaffolding would significantly assist in medical treatments. However, clinical applications of silk-based scaffolding are still underway and need clinical trials before official approval from regulatory bodies.

The results showed that mineralized silk still has a performance comparable to many natural and artificial fibers [138]. Moreover, the hybrid composite exhibited mechanical properties similar to bone in strength and modulus of elasticity. This makes silk superior to many other biomaterials used for tissue engineering in bones. Fine-tuning of the biomineralization parameters led to the controlled incorporation of hydroxyapatite onto native spider silk, maintaining good mechanical properties [138]. Natural bio-based nanomaterials can provide outstanding scaffold properties for bone tissue engineering [139] and cartilage regeneration [9,10].

### 6.3. Vascularization

The effectiveness of recombinant scaffolds and spider silk foams was even proved in the vascularization process, when a grouping of endothelial cells formed millimeter-branched beginnings of future blood vessels [108]. Considering this, the spider silk matrix has been used to develop artificial blood vessels [140]. The biomimetic design of structures with silk nanofibers has enabled constructs of vascular grafts with porous structures for blood vessel regeneration [141,142] or for small vascular grafts [143,144].

### 6.4. Ligament Repair

Advances in bioengineering research and orthopedic surgery have provided alternative solutions for the regeneration of musculoskeletal tissues of the human body [12,23,24]. Research shows that spider silk can withstand stresses similar to the ligament itself and even higher, which makes this biomaterial a good alternative for anterior cruciate ligament (ACL) of the knee replacement [134,145]. A biomechanical comparison was performed between an ACL composed of collagen fibers and an artificial spider silk construct. The ANSIS software was used to simulate the forces that occur during the anatomical movements of the ACL. The analysis showed that spider silk tissue outperforms ACL, thanks to its exceptional mechanical resistance, as it can withstand stresses of up to 2.5 MPa [145]. However, only experimental research still exists for the use of fibroin in ACL grafts, without clinical trials [25], even though research results are very promising [146]. Scaffolds made of silk that exhibit osteogenic function, interference screws, and tunnel fillers have been prototyped, with further research related to the stimulation of signaling pathways [25].

### 6.5. Muscle Tissue Repair

Musculoskeletal tissue engineering has benefitted from silk-based materials [12,23]. Nanofibrous scaffolds made from a mixture of silk, PLA, and collagen induce enhanced the adhesion, proliferation, and maturation of myoblasts. Recombinant spider silk proteins have been constructed in film form to examine their effect on rat cardiomyocytes. Cardiomyocytes cultured on eADF4 (k16) films successfully responded to extracellular stimuli and properly propagated electrical impulses. Apart from the fact that these films enable the adhesion of the most important types of cells in the heart tissue, they are non-cytotoxic and do not cause pharmacological or hypertrophic effects [147]. In addition, cardiomyocytes exhibited a higher expression of connexin 43, the protein responsible for the propagation of electrical impulses between cells [147]. It is challenging to design fully biomimetic muscle tissue together with the alignment of cells and adequate tissue responses. Nanofibers with silk fibroin have been studied for simple biomimetic skeletal muscle structures [148]. Conductive biomaterials can more efficiently promote muscle tissue growth [128,149,150]. Composite structures and blends, such as combinations of silk fibroin and gelatine, have been studied for skeletal muscle tissue engineering, including the design of structures that can be further used in flexible electronics and medical diagnostics [151].

### 6.6. Repair of Peripheral Nerves

**Figure 8:**
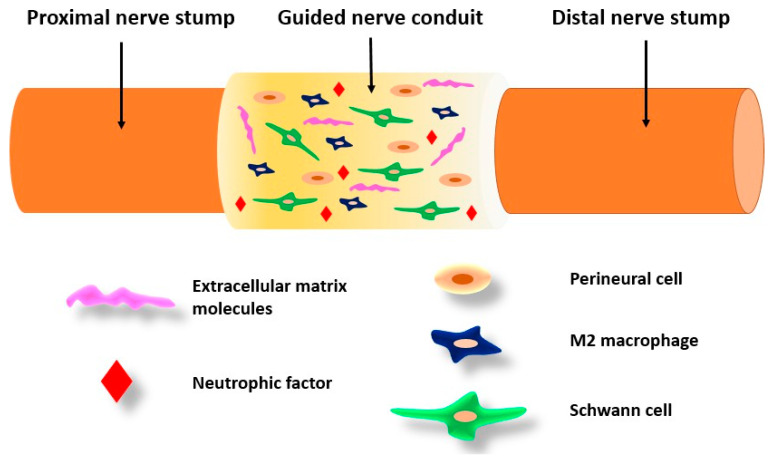
Peripheral nerve tissue engineering.

Spider silk has a proven capacity to guide cell proliferation and migration and enhance peripheral nerve regeneration [152]. Axon regeneration in peripheral nerve damage can be promoted by implanting specific biodegradable guidance channels which are able to guide the cells while present. Spider silk fibers were suitable for the human neuron culture and a study showed their very good adhesion, cell body migration, differentiation, and neurite (axon) extension, resembling ganglion structures [63]. After ten months, axons were regenerated with the presence of myelination, thus indicating that Schwann cells migrated through the constructs (Figure 8). Moreover, spider silk impregnated with collagen fibers is successful in cell differentiation and neural network formation. Neuronal cells were fully capable of activating action potentials, and in them, there was an increased expression of SNAP-25 protein, which is an indicator of the existence of functional synapses [153].

In addition, cultured neural progenitor cells, which have the potential to transform into neurons, astrocytes, and oligodendrocytes, showed successful differentiation on recombinant spider silk [154]. The most significant limiting factor for regeneration is the length of the gap. However, these nerve channels have been successfully applied in the peripheral nervous systems of animals [63].

## 7. Spider Silk in Nanomedicine

Unlike silkworms, the breeding of spiders is quite limited due to their cannibalistic and territorial nature. Although considerable efforts have been made to produce artificial recombinant spider silk, insufficient understanding of its nanostructure has prevented commercial success [155]. Nanomaterials are natural, accidentally obtained, or manufactured materials ranging in size from 1 nm to 100 nm, and the smallest changes at the nano level result in dramatic changes in the macro world [156].

Nanomedicine has emerged, offering potential solutions to the treatment of different conditions in relation to using silk-based materials, such as osteoarthritis treatment [129], or Achilles tendinopathy [157]. Research with gold nanoparticles in silk hydrogel used as a media for the laser treatment of subcutaneous bacterial abscesses has shown promising results for this complex medical condition [158].

### 7.1. Drug Delivery Systems

Soft porous natural materials represent very suitable materials for drug delivery systems, and silk-based biomaterials have shown different possibilities for tailoring such systems [3,91,103,109,125], including in the scope of bone tissue engineering [8]. Silk fibroin has shown good adjustability to suit different drug delivery systems, including hydrogels [116,117,121] or coatings and thin films [103]. Bioactivity and in vivo responses of silk proteins are important properties of the silk-based materials, utilized in this sense for tissue growth and remodeling [29,135]. It is also important that silk fibers can be used in the design of nanomaterials for drug delivery systems [159].

**Figure 9:**
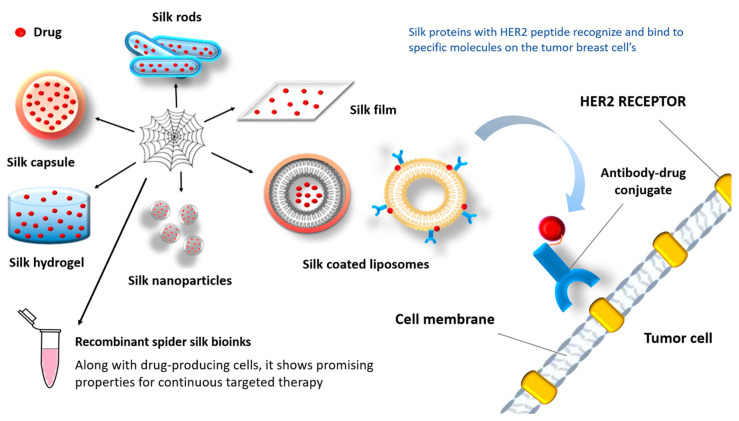
Silk-based biomaterials for chemotherapeutic delivery.

As is known, the biggest obstacle for an anticancer drug is controlled release at the target destination. Considering the characteristics of this material, the use of silk products (such as films, hydrogels, capsules, or liposomes coated with silk proteins) can potentially overcome that problem (Figure 9) [7].

Although surgery, radiotherapy, and chemotherapy have achieved substantial progress in cancer therapy, recent research has been focused on systems for the targeted delivery of drugs (therapeutics, hormones, inorganic nanoparticles, etc.) [160]. Silk represents an excellent tool in cancer therapy due to numerous advantages, including biocompatibility, biodegradability, the possibility of varied shaping, storing in a dried state [161], and the absence of systemic toxicity [162]. The basis of all systems for targeted drug delivery are nanomaterials constructed in the form of liposomes, micelles, dendrimers, and nanoparticles [163]. This type of treatment ensures a remarkably higher concentration of active substances in tumor tissues [164]. Moreover, these systems are naturally removed from the body due to enzymatic degradation (without harmful byproducts) [165]. The smaller the proportion of the crystalline phase (β-sheet), the faster the degradation occurs [166].

Increased selectivity is achieved by combining silk proteins with peptides (e.g., F3, Lyp1, CGKRK, or HER2 peptide) that recognize and bind to specific molecules on the surface of tumor cells (Figure 9) [167,168,169]. Since silk is hydrophobic, hydrophobic drugs give better results in combination with this biomaterial [170]. Also, negatively charged molecules are released faster than positively charged molecules [171].

Silk-based drug delivery systems can be applied locally or systemically [172]. For the needs of local delivery, various two-dimensional and three-dimensional systems are used. Two-dimensional structures include thin films, and coatings, made of silk fibers, while three-dimensional implants include hydrogels, foams, and porous scaffolds. Most of these structures have demonstrated the ability of sustained drug release over four weeks [172], inhibiting tumor growth in vivo [173]. Local delivery also involves transdermal methods that include microneedles for non-invasive and painless drug release.

This approach allows drug release by swelling and dissolution after passing through the skin [174]. Silk fibroin microneedles have been developed as electro-responsive material for specific drug delivery (insulin) [175]. Hydrogels that can have bioactive responses to external stimulus have become of great significance for the development of drug delivery systems and medical diagnostics [121].

Systemic drug delivery involves capsules [176], spheres [177], and particles containing active substances, which are released by diffusion or degradation of the material. Nanosized systems can reach the smallest capillaries and then be incorporated into cells through physiological barriers, which is crucial in cancer therapy. With this in mind, silk-based nanoparticles have been designed to deliver chemotherapeutics in tumor tissues [178].

Some of the techniques for producing silk nanoparticles include:

- Desolvation [179];
- Electrospraying [180];
- Ionic liquids [181];
- Laminar jet break-up [182];
- Microemulsion [183];
- Microfluidics [184];
- Milling technologies [185];
- Salting out [186];
- Self-aggregation [187];
- Sol-gel techniques [188];
- Supercritical fluids [189].

One way of controlling drug release using spider silk is thanks to pH-dependent carriers. Specifically, silk in combination with iron oxide nanoparticles [190] results in a limited release of the drug into the blood, that is, an increased release of the drug in the tumor tissue, which represents a more acidic environment (due to increased metabolic activity, more glucose brakes down and a large amount of lactic acid is produced) [191]. Moreover, tumor tissues are not homogeneous (they consist of different types of cells), so drug carriers can be modified to target the cells of the tumor microenvironment [172].

Septic arthritis is a medical condition that causes inflammation in the joints, bones, and cartilage. It is caused by a type of bacteria called *Staphylococcus aureus*, and currently available antibiotics are becoming less effective due to bacterial resistance [192]. However, scientists have successfully created a conjugate using spider silk and a thrombin-sensitive peptide (TSP) to deliver the antibiotic vancomycin directly to the affected area. Spider silk was dissolved in a MES (4-(N-morpholino)ethanesulfonic acid) reaction buffer. Then, 100 mM EDC [1-ethyl-3-(3-dimethylaminopropyl) carbodiimide hydrochloride] and 200 mM NHS (N-Hydroxysuccinimide) were added to activate it, which enabled the chemical binding of TSP. Finally, the encapsulation of vancomycin was carried out using the method described in the paper available at [193].

This conjugate has the potential to trigger drug release in the presence of specific enzymes produced by *Staphylococcus aureus* bacteria. Drug release from the conjugate particles amounted to 84.4% after 24 h of incubation, while plain spider silk particles only released 15.6% of the drug. Additionally, bacterial cultures were obtained from rat knee joint synovial fluid. The bacterial culture treated with the conjugate particles had an average of 40 CFU/mL, whereas the culture treated with plain silk had an average of 810 CFU/mL. This confirms the effectiveness of the conjugate particles in triggering drug release in the presence of infection [194].

### 7.2. Nanocomposites and Biomimetics

With the advancement of technology, the production of nanocomposites and nanomaterials inspired by spider silk or using silk-based materials has been investigated through different approaches [106,195,196]. For instance, to create a material with high stiffness, strength, and toughness, scientists combined silk with nanocellulose. The results showed that this composite could replace plastic (ecological importance) and serve as a basis for fabric production, even in medical implants [197]. In addition, spider silk was reinforced with graphene microparticles and carbon nanotubes, resulting in the strongest known fiber [198]. However, not only the structure of individual spider silk fibers was considered. Here, the design of the entire spider’s web served as a template for different devices and applications, which is the subject of research in biomimetics. Silk-based microspheres can be added to the cell cultures to enhance cell growth and adhesion [199]. The biomimetic approach in material design has opened up many new directions in material structures, as well as final applications [129,141] and including adjustments of existing fabrication technologies for silk fibers [30,200].

In bone tissue regeneration, the development of new nanocomposites, and especially those derived from nature, has opened new research directions [148,201]. Combination with different nanoparticles, such as silver nanoparticles, can enhance antibacterial effects [202]. The combination of titanium dioxide nanotubes as drug carriers with zeolite-based compounds and silk fibroin has shown promising properties for drug delivery systems [203]. Silk fibroin in composite structures can enable multifunctional drug delivery microcarriers [159,204].

Combinations of silk fibroin with hydroxyapatite and/or graphene oxide as nanocomposites have enabled the construction of porous scaffolds with very good mechanical properties and improved capabilities for bone tissue regeneration [205]. Nanocomposite structures have also been studied to enable the design of in vitro models for cancer treatments in bone tissues [206].

Spider silk, as one of the silk variations, has been used in many applications, including biomedical ones. It has been studied and analyzed, with clear potential proven in experimental lab studies, towards smart biomaterials and novel composite structures that can mimic natural tissues. However, clinical applications are rather limited to the specific areas of cosmetics, wound dressing, breast reconstruction, or certain other treatments, and it is almost without use in musculoskeletal tissue engineering. Common biomaterials considered for bone tissue scaffolds are focused on those that can primarily provide mechanical strength. New development directions towards smart or biodegradable structures should encounter less-acknowledged materials like silk-based ones for load bearing applications, especially considering that the tailoring of properties within composite structures can be achieved. In such applications, spider silk and silk fibroin can provide additional properties that are not fully exhibited with currently used biomaterials, beside a very important sustainability. However, production costs are still very high and challenging and further research focus should be directed toward discovering or upgrading fabrication procedures to enable the wider availability of spider silk.

## 8. Conclusions

This review presented the latest research related to the potential applications of spider silk in reconstructive and regenerative medicine and tissue engineering, including nanomedicine and drug delivery systems, with a focus on musculoskeletal tissues. Spider silk is a natural material with long-established use in different applications. Silk-based materials have emerged as sustainable, natural materials that can provide significant benefits in the development of advanced biomaterials for medical implants and tissue engineering. Bone and cartilage, muscle, and tendon tissue engineering, as well as the advanced design of skin and vascular tissues, can greatly benefit from bioactive and smart biomaterials, incorporating silk-based biomaterials. Silk proteins in bioactive materials that mimic the tissue structure can enhance tissue regeneration and growth. Silk proteins have shown good adjustability to suit different drug delivery systems.

Natural spider silk synthesis and the further recombinant production of spider silk proteins have been reviewed. Silk-based biomaterials used for tissue engineering applications (scaffolds, hydrogels, films, fibers, or nanoparticles in drug delivery) have been successfully designed and developed for various tissues such as bones, tendons, ligaments, skin, muscles, and nerves. However, the use of natural spider silk remains limited due to low production yields and difficulties in cultivation. Hence, the only practical solution is the production of recombinant spider silk proteins.

Preliminary research results and tissue engineering examples with silk-based materials showed no inflammatory reactions, but in vivo studies of spider silk-based materials remain limited. The degradation of fibers by macrophages confirmed the property of biodegradability, and consequently the newly grown tissue can replace the silk material. Furthermore, the by-products of silk degradation are non-toxic, which means they can be recognized and neutralized by the immune system, unlike many synthetic polymers. In this regard, another important characteristic is the rate of degradation. Spider silk materials remain mechanically stable for a significant time, without increase in brittleness or susceptibility to tearing under physiological conditions, which is a desired material property in neurological-related applications. Bioactive bone grafts with conductive properties for signaling pathways are the latest research direction promising great advancements.

Silk proteins are important in designing tissue-on-chip or organ-on-chip technologies and micro devices that have started to be used for the precise engineering of artificial tissues and organs, disease modeling, and the further selection of adequate medical treatments. Recent research indicates that silk (films, hydrogels, capsules, or liposomes coated with silk proteins) has the potential to provide controlled drug release at the target destination.

The specific properties of spider silk material show some clear advantages and disadvantages. However, a comprehensive understanding of its nanostructure and associated mechanisms in bioactive material systems is needed, as well as further clinical trials to gain approval for its use in tissue engineering.

## Data Availability

No new data were created or analyzed in this study. Data sharing is not applicable to this article.
